# Gender and blindness: Taking a global and a local perspective

**DOI:** 10.4103/0974-620X.53032

**Published:** 2009

**Authors:** Paul Courtright

**Affiliations:** Kilimanjaro Center for Community Ophthalmology, Moshi, Tanzania

Our understanding of gender and blindness started with a systematic review of global population-based blindness surveys carried out between 1980 and 2000. This systematic review and metaanalysis showed that blindness is approximately 40% more common in women compared to men, regardless of age.[1] The imbalance was mostly notable among people above 50 years of age, with women consistently having higher prevalence figures compared to men.

The meta-analysis stimulated investigations into the reasons for the excess prevalence and there have been a number of studies since then to explore possible explanations. These studies have ended up showing the role of gender (social constructs that define roles of men and women in society), rather than sex (biological differences between men and women) as primarily responsible for the female excess. The data from surveys of cataract surgical coverage (CSC) prior to 2001, showed lower CSC rates in women compared to men, in every study undertaken.[2] Recently, the CSC data was updated (2000–2008), revealing that lower CSC in females has persisted, even since the launch of Vision 2020, a global initiative launched jointly by the World Health Organization and the International Agency for the Prevention of Blindness.[3] If women received cataract surgery at the same rate as men, global cataract blindness could be reduced by approximately 11–12%.

Global data on trachoma has also revealed a preponderance of women with trichiasis (and associated vision loss) compared to men.[4] This data has been summarized recently with a systematic review and meta-analysis, similar to the previous work with blindness surveys. The meta-analysis revealed that globally women have a 1.8-fold higher risk of trichiasis compared to men; put another way, in the surveys, women accounted for 70% of all patients with trichiasis.[5] While this information is useful in understanding the burden of trichiasis for both men and women, the absence of trichiasis surgical coverage data, disaggregated by sex, limits our knowledge of whether women have equal access to surgical services compared to men.

Analysis on gender from other eye conditions has been infrequently carried out, limiting our understanding to only a few settings where data, often reported more as an aside, suggests that gender inequity exists in use of services. Particularly, glaring is the lack of understanding of possible gender inequities in use of services for diabetic retinopathy, nontraumatic pediatric cataract, and glaucoma.

Can we extrapolate the findings from the global assessments to the local setting? Yes, and no. When the original blindness prevalence meta-analysis was carried out, there was no prevalence data from Latin America or the former Soviet Union. The meta-analyses of cataract surgical coverage and trichiasis prevalence are limited by the places where studies were done. However, there is no indication that these studies were carried out due to an anticipated male:female disparity.

Oman has had the distinction of having quite a few well-designed population-based surveys, which can provide some local perspective. The national population survey during 1996–1997, revealed that women had an age-adjusted prevalence of blindness of 1.4% compared to men at 0.8%; giving women a 1.89-fold (95% CI, 1.55–2.51) higher risk of blindness compared to men.[6] Using the same database, the researchers found that men (with cataract) were 1.55-fold (95% CI 1.50–1.60) more likely to have received cataract surgery compared to women.[7] These findings are similar to the global estimates and, as the authors note, health education directed at women and improved access to surgical facilities should help to reduce inequities. In the eight years between the 1997 survey and the 2005 survey, a 29.3% reduction in blindness was detected, but women continued to have a statistically higher prevalence of blindness (9.93% among 40+ age) compared to men (6.59% among 40+ age).[8] It will come as no surprise that the 2005 survey found an age adjusted 1.79-fold higher (95% CI 1.32–2.42) prevalence of trachomatous trichiasis in women compared to men.[9] Suggestions for addressing gender inequity focused on efforts at the community level (women′s groups), as well as, at the health provider level (providing counseling and better screening). No sex difference was detected in the prevalence of glaucoma, the fourth leading cause of blindness in the 2005 Oman Eye Study.[10]

Addressing gender issues in blindness is a global and a local issue; identifying inequity is the first and the most critical step. Identifying the reasons for inequity and strategies to address inequity are the next logical steps. Oman has progressed further than most countries in identifying sex-specific differences in blindness and various eye conditions. This puts, Oman ahead of other countries in terms of addressing gender inequity and the World Sight Day theme for 2009, "Gender and Eye Health", will be a tremendous opportunity to celebrate successes and tackle the challenges of the future. Making VISION 2020 programs, globally and in Oman, more responsive to changing healthcare needs and the demographic environment, is good for both women and men.
